# Dental Hygiene

**Published:** 1876-04

**Authors:** D. C. Hauxhurst


### DENTAL HYGIENE.

BY D. C. HAUXHURST.

CONTINUED.

*Report to the Michigan State Dental Association.*

It is in the mother's womb that the foundation of every set of teeth is laid. The young pulps, charged with nutritive juices from the mother's blood, unfold and develop while subject to whatever physiological or pathological condition may chance to exist in her organism. And if the tissues of the teeth are organized badly in these early months, they will be subject to decay in after life. The following are some of the unfavorable intra-uterine influences:

1. The development force that governs the distribution of molecules and the building of cells in the young tissues may be deficient in quantity, or abnormal in its action, on account of the pathological states of the mother.

2. Or, some casual agency may come from without like a shock upon the growing organ, transmitted by the mother's organization, determining minute defects of structure. Such structural defects have been described by many careful observers.

3. The pabulum supplied to nourish the young teeth-germs may be deficient in quality or quantity. A deficiency in mineral salts in the mother's blood is believed to be a common cause of arrested development or imperfect development of the dental tissues of the embryo.

Either or all of these causes may act, or others not enumerated. Much might be said under each head, but that would be foreign to my present purpose.

Whatever agencies are at work in a given case should be resolutely faced and removed by the practitioner, And the prospective mother should be instructed in whatever liabilities exist, and well fortified in such prophylactic measures as are possible.

Intelligent women have already began to call upon the dental physician to tell them how to provide for the sound growth and right organization of their unborn infant's teeth. As his own knowledge of the phenomena and laws of early dental development increase, the dentist will be the better able to meet this demand with right teaching.

I look forward to the time when every mother shall be able to know how to endow her offspring with soundly organized teeth, even though she may not be able always to se cure the requisite conditions.

But while we are caring for the teeth of the mother, will not her own teeth suffer through the wonderful sympathies that play between them and the gravid uterus? May they not suffer on account of the abstraction of lime-salts from the blood to supply the heavy demands of the rapidly developing fœtus? Although most of the viscera have sympathies with the teeth through the ganglionic nerves, yet none of these sympathies are of such commanding importance as those that play between the teeth and the uterus.

We have pregnancy tooth aches that are lawless and seemingly without cause, acid secretions and a disordered mucous membrane in the oral cavity, irregular pains affecting the nerve distribution of the gums, and even neuralgias about the face and head. Much trouble often arises from lesions the most trifling and such as at another time would cause no irritation. These sympathies are more common from the second month of pregnancy up to the sixth.

The secretions often become acid and rapidly attack the teeth. A greater destruction will be wrought within these limits than during years of time when the uterus is not gravid. All these symptoms must be met by the practitioner and if possible held in abeyance until pregnancy is over. He must be equal to the occasion and save his patient's teeth. And for this purpose he will advise the strictest cleanliness, alkaline washes with abundant and frequent rinsings of mouth and teeth; he will often scrutinize their contact surfaces; he will advise air, sunlight and exercise as may be required; remembering always the stomach and even addressing a wise medication to this organ if required. He will advise the use of unbolted flour or other foods that shall furnish the blood reservoirs with the phosphatic salts. Calcic phosphate in particular is abundantly and rapidly withdrawn from the mother's blood during pregnancy to supply the developing osseous system of the child and unless there be a daily supply in the mother's food her own teeth as well as the rest of her tissue must suffer.

I do not dwell upon this point; I make but the merest allusion to a great subject that could scarcely be well opened up in twenty lectures. What I wish to impress, is that the dental practitioner must qualify himself to save his patient's teeth whatever her condition and whatever remedial or hygienic agencies her case may demand.

These will be various and far more numerous than I have even hinted at.

